# Role of Senescent Cells in Cutaneous Wound Healing

**DOI:** 10.3390/biology11121731

**Published:** 2022-11-29

**Authors:** Allison M. Andrade, Mingda Sun, Nathan S. Gasek, Geneva R. Hargis, Roshanak Sharafieh, Ming Xu

**Affiliations:** 1 UConn Center on Aging, UConn Health, Farmington, CT 06030, USA; 2 UConn School of Medicine, UConn Health, Farmington, CT 06030, USA; 3 Department of Surgery, UConn Health, Farmington, CT 06030, USA

**Keywords:** aging, cellular senescence, heterogeneity, wound healing, acute cutaneous wounds, chronic cutaneous wounds, p16, p21, senolytics

## Abstract

**Simple Summary:**

The biology of cellular senescence has broad implications for the fields of aging, tissue and wound repair, tumor biology, and development. Cellular senescence is a state of irreversible growth arrest, which is induced by internal and external stress mechanisms. Senescent cell populations are diverse and display transcriptomic and biomolecular variability, making it difficult to find a sole specific biomarker from defining this state. Although this cell fate is involved in cutaneous wound healing and tissue repair, there is still a substantial gap in understanding how senescent cells modulate regenerative processes. Addressing remaining key questions in the field may guide clinical care and management of both acute and chronic wounds and the development of novel therapeutic agents.

**Abstract:**

Cellular senescence has gained increasing attention in the field of aging research. Senescent cells have been implicated in biological aging processes, tumorigenesis, development, and wound repair amongst other processes and pathologies. Recent findings reveal that senescent cells can both promote and inhibit cutaneous wound healing processes. Relating senescent cells in acute and chronic wounds will help to clarify their role in wound healing processes and inform our understanding of senescent cell heterogeneity. To clarify this apparent contradiction and guide future research and therapeutic development, we will review the rapidly growing field of cellular senescence and its role in wound healing biology.

## 1. Introduction

Biological aging encompasses the cellular and molecular mechanisms behind changes in the body and the resulting pathologies associated with chronologic age [1]. Cellular senescence is increasingly implicated with the process of biological aging. In particular, chronologically aged and dysfunctional tissues are associated with the accumulation of senescent cells [2]. In addition, senescent cells have been linked to tissue damage and tumor proliferation [2,3,4]. Though many studies have highlighted the pathologic roles of these cells, there is increasing interest in their beneficial roles to promoting homeostasis. Thus, the senescent cell fate has also garnered interest for its roles in tissue repair, tumor suppression, as well as embryogenesis [2,3,4]. 

Tissue damage and repair, or wound healing, highlights the duality of cellular senescence. Aging and tissue damage are closely related, as age-related pathologies usually involve perturbations in the underlying tissue. Additionally, most chronic wounds arise from age-related pathologies [5]. A more comprehensive understanding of how senescent cells contribute to wound healing will allow for the development of novel therapeutics to address limitations of current treatment modalities for chronic wounds. In this review, we provide an overview of cutaneous wound healing and mechanisms of cutaneous tissue repair to model the role senescent cells play in tissue healing. 

We will first discuss cellular senescence and characteristics of senescence cells and then provide an overview of wound healing processes with an emphasis on cutaneous wounds. Finally, we explore how cellular senescence is implicated in the wound healing process and discuss cellular senescence as a therapeutic target for wound healing.

## 2. Cellular Senescence

Cellular senescence is a cell fate defined by a permanent arrest from the cell cycle [2,3,4,6]. This cell state was first described in the 1960’s in human diploid cells that had undergone serial passages to the point of non-division [7]. Beyond replicative stress, senescence is also elicited by other stressors to the cell such as metabolic, oxidative, and oncogenic stress [2,3,8]. Additionally, such stressors can provoke telomere shortening, DNA damage pathway activation, mitochondrial dysfunction, and oncogene induction [2,3,8]. Morphologically, senescent cells are larger than non-senescent counterparts and demonstrate enhanced granularity associated with their altered metabolism and increased lysosomal content [2,3,8]. At the transcriptomic level, there are also widespread changes in gene expression within senescent cells which have been reviewed previously [4,9]. A great deal of interest in the field has been inspired by findings linking cellular senescence to a number of age-related pathologies as well as biological aging itself [2,4,8]. In particular, there is evidence that the removal of senescent cells in an organism may lead to an increase in health span and lifespan, and conversely that the addition of senescent cells to a previously healthy organism may increase overall morbidity and mortality [2,10]. 

## 3. Senescence Characteristics

Senescent cells are heterogeneous, and currently there is no single marker that can define a cell as senescent [2,8]. Furthermore, these cells produce a typically pro-inflammatory secretome referred to as the senescence associated secretory phenotype (SASP), which possesses corresponding complexity [2,4,8,11,12]. The SASP is responsible for paracrine and endocrine signaling via chemokines, cytokines, growth factors, and proteases that are all released from the senescent cells and can signal other neighboring cells into a state of senescence termed “paracrine senescence” [2,13]. 

There are a number of ways that senescent cells can be detected experimentally. Several of these techniques rely upon increased organelle dysfunction present in senescent cells. Mitochondria accumulate in senescent cells due to their altered metabolism [2,9]. Reactive oxygen species (ROS) production, redox state, mitochondrial function, and mitochondrial biogenesis assays can peripherally inform investigators of senescent cell burden [2]. Lysosome accumulation is another common indicator of cellular senescence [2,9]. Two markers of senescence in the lysosome include lysosomal senescence-associated beta-galactosidase (SA beta-Gal) and lipofuscin [2,9]. Both of these markers can be measured using an activity assay, which is a simple test to profile senescent cells [2,9]. Along with mitochondrial and lysosomal overabundance, the nucleus is also affected by the activation of a senescent state [2]. DNA damage response pathways lead to activation of the cyclin dependent kinase inhibitors p16 and p21 and subsequent cell cycle arrest. Other nuclear markers include phosphorylation of histone H2AX and the related telomere associated foci [2,9]. DNA Segments with Chromatin Alterations Reinforcing Senescence, or DNA-SCARS, are another type of DNA damage found within senescent cells referring to persistent damage of DNA marked by DNA damage response proteins [2,9,14]. DNA-SCARS are also called senescence-associated DNA damage foci, and are associated with growth arrest and interleukin 6 (IL-6) secretion in senescent cells [14]. RNA sequencing of senescent cells reveals that SASP-associated gene expression presents an additional layer of heterogeneity [8]. Sequencing data reveal differential gene expression of senescent cells dependent on stressor type, cell type, and duration of senescent state [15]. A new algorithmic technique that involves two phases of testing is under development for defining a senescent state via detecting multiple hallmark characteristics [16]. Phase one of the assessment validates the cells are in a senescent state by testing markers such as: SA-B-Gal or lipofuscin, p16, p21, lamin B1, and SASP proteins [16]. After phase one defines the presence of senescence, phase two allows for characterization of the senescence subtype by testing for transcriptomic features, for example pro-inflammatory SASP transcripts, as well as secreted proteins [16]. 

The heterogeneity of cellular senescence poses a distinct challenge because there is not one common defined marker that is present in every senescent cell [2,8,17]. There has been some success in identifying several common markers in senescent cells including activation of the p16/retinoblastoma and the p21/p53 signaling pathway, as mentioned previously [2,8]. Due to this, p16 and p21 gene expression and protein levels are common indicators that a cell is in a senescent state [2,4,18,19,20,21]. Other markers include p19, uPAR, and glycoprotein non-metastatic melanoma protein B (GPNMB) [8]. While both p16 and p21 expression are common markers leveraged by the field, it is important to note their limitations. In particular, these proteins may be transiently upregulated in diverse biologic contexts. Examples of their “non-senescent” upregulation include p21′s roles in embryologic development and hair growth cycles, or the presence of p16 in embryonic tissues and basic macrophage physiology [8,17,22,23,24,25]. Additionally, the accumulation kinetics of these proteins vary during the initiation of a senescent state [26]. A study using a radiation-induced osteoporosis model suggests that p21 is upregulated when the cell first becomes senescent, while later p16 activation maintains the senescent state [3,26]. However, this work also suggested that the individual roles of p21 and p16 appear to be independent of one another in the context of the model system used [26]. Bulk RNA sequencing data from human fibroblast and mouse fibroblasts shows a difference in transcriptomic signatures and SASP products based on senescence inducer, cell type, and stage of senescence [8,15]. To further validate the heterogeneity of senescent cells, a study revealed that different senescent cell inducers (oncogenic stress, replicative stress, IR-induced, and Dox-induced) applied to the same cell line evoke differential RNA expression supporting that senescence is a diverse state depending on cell lineage and stress type [27]. Furthermore, this heterogeneity is recapitulated at the single cell resolution as demonstrated by the variation of transcriptomic signatures of senescent cells found within individual cultures of human fibroblasts subjected to identical culture conditions [8,28]. As cellular senescence may be elicited by different stressors across environmental contexts, multiple markers should be tested when assessing SASP production in senescent cells [8,17].

## 4. Cutaneous Wound Healing

**Figure 1:**
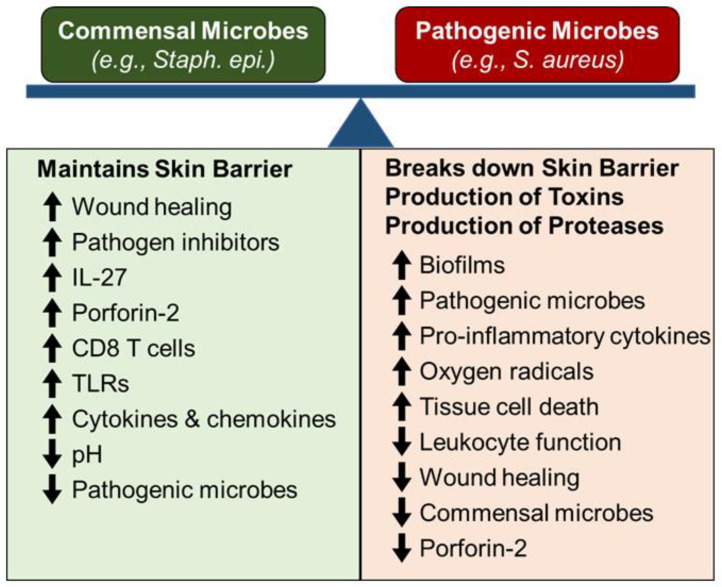
The skin microbiome balance and skin wound healing.

One of the primary functions of skin is to provide an environmental barrier, which creates a sterile environment for the underlying cells and connective tissue. Central to maintaining this barrier is the balance of colonization between commensal (non-pathogenic) microbes (e.g., *Staphylococcus epidermidis*), and pathogenic microbes (e.g., *Staphylococcus aureus*) [29,30,31,32]. Commensal microbe colonization of skin maintains skin barrier function by suppressing pathogenic microbe colonization and promoting low-level/baseline innate immunity on local skin to monitor and quickly respond to any barrier breaches (Figure 1). Alternatively, colonization of skin by pathogenic microbes, destroys the architecture and function of the skin, by releasing microbial toxins and proteases that directly breakdown key skin barriers (e.g., keratinocytes and connective tissue), as well as inducing acute and chronic inflammation, which in turn, destroys the cells and matrix in the skin (Figure 1). Additionally, mechanical injury (e.g., abrasions, cuts or burns) to the skin also breach the skin barrier and promote both pathogenic microbe colonization, as well as tissue destructive acute and chronic inflammation, all of which can compromise wound healing [32,33,34,35]. Thus, controlling skin microbe colonization, mechanical injury of skin, and related tissue reactions (inflammation and wound healing) is key to both maintaining and re-establishing effective skin barrier function. Since inflammation and wound healing are central for maintaining and re-establishing skin barrier function, it is important to understand these processes, as well as the impact of aging on their function in both normal and aging skin [17,29,31,32,33,36,37,38,39].

**Figure 2:**
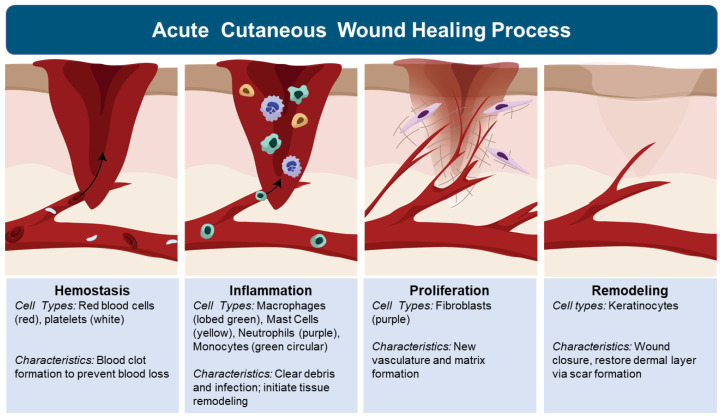
Acute cutaneous wound repair occurs in four well defined stages: hemostasis [6,33,35,39], inflammation [6,33,35,39,41], proliferation [40], and remodeling [6,32,34,42], which are mediated by distinct cell populations.

Tissue response to injury, regardless of organ, begins with inflammation. The inflammatory response is triggered to protect the body by localizing and eliminating the damaged tissue to allow the body to heal. The inflammatory phase of tissue response to injury, is activated by skin mast cell release of vaso-active amines with increased vaso-permeability of blood plasma (edema) within the injured tissue [6,33,35,39]. This initial wave of fluids to injured tissue provides immediate clot formation (Figure 2). Besides preventing further bleeding, the clot serves as a temporary tissue matrix to stabilize the injury and presents a barrier to the environment, including pathogenic microbes [6,33,35,39]. This movement of fluids is followed by a surge of leukocytes, which include neutrophils, followed by macrophages and lymphocyte infiltration (Figure 2) [6,33,35,39,40]. Neutrophils function as the first line of defense, killing pathogens and clearing cellular debris [40]. Monocytes circulate in the bloodstream and differentiate into macrophages at the wound site where they phagocytose dead neutrophils, pathogens, and tissue debris and release pro-inflammatory cytokines (e.g., IL-1, IL-6, IL-8, and TNF-α) that can sustain or further amplify the inflammatory response to resolution or to chronic inflammation leading to tissue destruction [41]. Central to determining these outcomes are macrophage subpopulations, including M1 and M2 macrophages [6,33,34,35,37,39]. M1 macrophages promote inflammation which clears injurious agents but can also destroy tissue structure and function. M2 macrophages promote wound healing by stimulating keratinocyte, fibroblast, and endothelial cell proliferation and migration, which assists wound transition to the proliferation phase [41]. During the proliferation phase, fibroblasts weave collagen fibers to repair tissue structure, and provide matrices for cell migration to occur (e.g., endothelial cell migration needed to form new vasculature to sustain the healing wound tissue) (Figure 2) [40]. The formation of granulated tissue signals an important transition, in which keratinocytes migrate across the newly formed matrix to close the wound [40]. Although the migration of keratinocytes does restore the epidermis layer of skin, the architecture and function of the dermis is lost with scar formation (Figure 2) [6,33,35,39]. This cascade of tissue responses is central to understanding and controlling cutaneous inflammation and wound healing. Although there is significant information that exists related to inflammation and wound healing in the skin of healthy individuals, the impact of aging and roles senescent cells play on inflammation and wound healing, is only beginning to be unraveled. 

**Figure 3:**
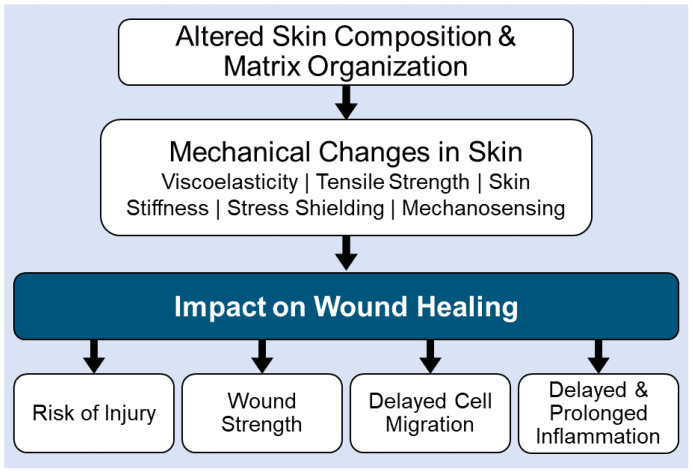
Impact of aging on skin composition, function & wound healing [6,34,38].

Aging can impact the skin microbiome (e.g., commensal vs. pathogenic microbe colonization) by changes to the skins’ structure and function. For example, alterations in hormonal, metabolic, and/or immune systems, increased wrinkle formation, decreased elasticity, defective wound healing, decline in the production of sebum, and decreased water content, can alter microbe colonization patterns in the skin [6,17,29,30,31,32,34,35,36,37,38,39]. Age-related loss of effective immunity in the skin, promotes the colonization of pathogenic microbes, which in turn promote chronic inflammation and excessive tissue destruction in the skin. Unfortunately, aging also negatively impacts the cells that are critical to tissue remodeling by altering skin architecture and function (Figure 3). This includes the accumulation of senescent tissue cells (keratinocytes and fibroblasts), decreased cell migration capabilities, modified extracellular matrix remodeling with diminished fiber density and increased matrix fragmentation, loss of elastin networks, and glycosaminoglycan alterations. These age-related changes in the skin architecture, alter viscoelasticity, tensile strength, skin stiffness, stress shielding, and mechanosensing [6,17,29,30,31,32,34,35,36,37,38,39], all of which lead to defective wound healing in the aging populations (Figure 3). 

## 5. Chronic vs. Acute Wounds

While acute wounds generally follow the pattern of events described above, chronic wounds feature alterations including changes in cellular composition, cellular localization, and transcriptomic profiles ultimately leading to delayed and pathological healing [6,43,44]. Chronic wounds experience prolonged inflammation, as compared to normal wounds, which generally spend less than two weeks in the inflammatory phase [45]. The prolonged inflammation is characterized by an excess amount of pro-inflammatory macrophages at the site of the wound [46]. Specifically, M1 macrophages in a chronic wound that are unable to transition into M2 macrophages disrupt the proliferation phase in chronic wound healing [46]. One study found that MiR-21 is increasingly upregulated in the macrophages of diabetic wounds as compared to acute wounds, indicating that MiR-21 may inhibit inflammation resolution [47]. Another cause of prolonged inflammation is due to the presence of bacterial biofilms leading to activation of more neutrophils and pro-inflammatory macrophages, which may further propagate senescence [48]. The presence of a bacterial biofilm on a chronic wound complicates treatment since some therapies are not effective at eradicating the entirety of the biofilm [48]. Understanding how senescent cells contribute to these mechanisms that drive inflammation may help create more effective therapies that limit a cellular senescence provoking positive-feedback loop.

Chronic wounds are a common comorbidity associated with type II diabetes and metabolic dysfunction [49]. According to the American Diabetes Association (ADA), over 9–12 million Americans suffer from chronic, non-healing wounds, presenting a serious health care and socioeconomic burden [49]. Chronic, sterile inflammation has been observed in diabetic wounds as a result of a heightened and dysfunctional immune response involving excessive accumulation and retention of immune cells, like macrophages and neutrophils, which release pro-inflammatory cytokines, cytotoxic enzymes, and free radicals [6,50]. Beyond changes in relative cell type abundance, single cell profiling suggests that there are distinct cellular subpopulations associated with wound chronicity as evidenced by enrichment for unique matrix remodeling fibroblasts in resolving compared to chronic diabetic wounds [44]. Hyperglycemia has been identified as one of the major causes of several diabetes-related comorbidities and the creation of environments of chronic, low-grade inflammation in wound healing [45]. Hyperglycemia causes heightened levels of ROS and advanced glycation end products, which can consequently result in abnormal extracellular matrix (ECM) function, delays in vascular regeneration and re-epithelialization, and wound hypoxia [43,45]. Hyperglycemia also promotes senescence and drives various senescence responses including the SASP [45]. For example, hyperglycemia promotes the expression of the *AQR* gene which induces senescence in endothelial cells [51]. As mentioned, chronic wounds are highly susceptible to developing bacterial biofilms and pathogenic infections that can lead to sepsis and death [6]. This would also explain the high progression rate of diabetic ulcers which tend to advance until the affected region may need to be amputated [50]. Moreover, in late stages of wound healing, diabetic ulcers are also at risk of scar hypertrophy, which may lead to development of keloid scars [50]. Collectively, promotion of cellular senescence in a chronic wound environment and the accompanying pro-inflammatory SASP may contribute to the impaired healing process.

## 6. Implications

The underlying biology of senescent cells and wound healing feature substantial overlap when considering the SASP [6]. The pro-inflammatory signaling cascades associated with the SASP provide a number of chemotactic cues that recruit immune cells such as neutrophils, monocytes, natural killer (NK) cells, T and B cells, as well as mast cells to the site of inflammation [6]. 

The pro-inflammatory phenotype is beneficial for immune cell recruitment in acute wound repair. Under homeostatic conditions, immune cells will clear senescent cells after acute wound closure [6,52]. Senescent macrophages within the wound bed of acute wounds release IL-6 as part of the SASP, which promotes cellular plasticity and repair [6,52,53]. This cellular plasticity promotes regeneration of the surrounding wound bed [53]. This pro-inflammatory phenotype serves a beneficial role in the early stages of tissue repair and wound healing, albeit when the process is well controlled and short lived [6,52]. Adverse effects are observed when the pro-inflammatory SASP is sustained within a tissue [3,52]. Prolonged SASP signaling can promote tumor development, chronic inflammation, and immune deficits [6,52]. The pro-inflammatory SASP can activate other neighboring and recruited immune cells into a state of cellular senescence as well [52]. This pro-inflammatory phenotype is observed in chronic wounds, wherein the immune cells that become senescent are unable to clear the pre-existing senescent cells which leads to further senescent cell accumulation [52].

In acute cutaneous wounds, senescent cells are observed as a part of the healing process and contribute to overall healing (Table 1) [6,52,54]. Senescent fibroblasts produce platelet-derived growth factor alpha polypeptide a (PDGF-A+) in the SASP to promote wound healing [54]. Additionally, senescent fibroblasts promote differentiation of non-senescent fibroblasts into myofibroblasts, contributing to wound contraction [54]. When myofibroblasts become senescent, they function as a limiting factor to fibrosis through their SASP, which works to degrade the ECM [55]. Similar to fibroblasts, endothelial cells will help promote myofibroblast senescence by PDGF-AA in the SASP, demonstrating that both fibroblast and endothelial components promote wound contraction and limit fibrosis [18,54]. Macrophages have various functions within the process of wound healing [42]. M1 macrophages are associated with a pro-inflammatory response in wounded tissues [42]. Polarization of M1 pro-inflammatory macrophages to M2 anti-inflammatory macrophages helps encourage debris resolution and tissue remodeling under physiologic states [42]. Of note, this balance of polarization states and resulting influence on wound chronicity may change in pathologic conditions such as diabetic foot ulcers wherein localized relative increases in M1 signatures are associated with wound resolution [44].

In aged and diabetic individuals, cellular senescence can promote wound chronicity and persistent inflammation. Multiple studies have shown the presence of senescent cells in both wounded and uninjured diabetic skin [6,45]. For example, changes in morphology and cellular processes characteristic of senescence have been observed in fibroblast cultures from diabetic ulcers and uninjured skin, as well as fibroblasts isolated from the ulcers of diabetic patients [45]. This includes an overexpression of p53/p21- pathway members, heightened levels of senescence-associated β-galactosidase (SA-β-Gal) activity, increased γ H2AX (pH2AX) levels indicative of DNA damage, and diminished proliferative capacity [45]. Evidence shows that although transient senescence produces growth factors to accelerate skin repair and prevent excessive fibrosis, chronic senescence is implicated in fibrotic disease [6]. Moreover, in other major cell types involved with wound healing such as keratinocytes and endothelial cells, elevated blood glucose levels have been shown to enforce senescent phenotypes and morphology [45]. Oxidative stress and dysfunctional mitochondria, which are prevalent in the diabetic environment, stimulate and perpetuate senescence [6,45]. Ultimately, diabetic wounds exhibit levels of tissue damage, dysfunction, and stress similar to aged tissues [45]. With aging, regeneration and wound healing are delayed with the accumulation of senescent cells (e.g., senescent keratinocytes) contributing to loss of regenerative ability [6,56,57,58].

**Figure 4:**
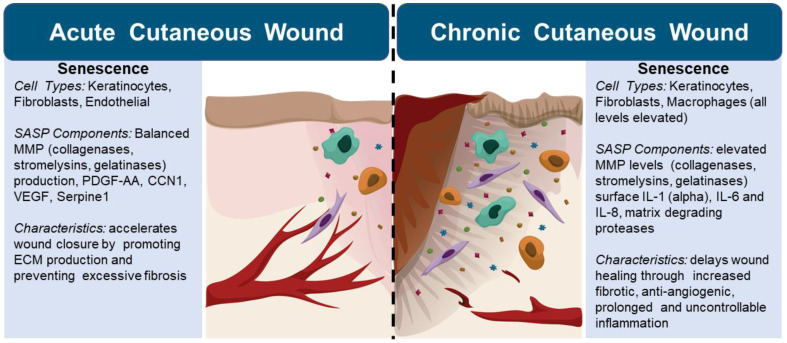
Senescent cells in acute and chronic cutaneous wounds [45]. Senescent cells vary by type, distribution, SASP production, and elicit varying phenotypes depending on wound chronicity [42,46,52,53,54,55]. Cellular senescence may participate in physiologic healing in acute contexts while inhibiting overall wound closure in a chronic setting [42,50,51,52,54,56,57,58,59].

Importantly, components of the pro-inflammatory SASP have been linked to heightened inflammation, immune cell accumulation, and dysfunction in senescent cells (Figure 4). Clinical studies have isolated a significant population of pro-inflammatory M1 macrophages in diabetic wounds, leading to the theory that senescent macrophages may be linked to sustained inflammation present in impaired wound healing [59]. During the wound healing process, macrophages exhibit polarity and plasticity [59]. However, it has been shown that the SASP prevents macrophage shift from M1 to M2, potentially contributing to the prolonged inflammatory phase of diabetic wounds [59]. Diabetic tissue has been found to harbor a variety of senescent macrophages that exhibit altered polarization and produce a CXCR2 associated SASP [45,60]. CXCR2 is a receptor that can be activated by SASP components, and its expression has been associated with nuclear induction of p21 [60]. Blocking signaling through CXCR2 is linked to improved wound healing in diabetic mouse models and ex vivo skin models [60]. Although macrophages have been shown to express SA-B gal, which is a common marker of senescence, it should be noted that lysosomes in macrophages naturally present B-galactosidase activity [45]. The overall role of cellular senescence in cutaneous wound closure is complex and context dependent as is exemplified when comparing acute and chronic wounds. Thus, it is essential to understand the heterogeneity of senescent cells and examine their roles in different wound types.

## 7. Practical Applications to Target Senescent Cells

Senescent cells can be pharmacologically targeted using senolytics. This putative drug class is designed to specifically eliminate senescent cell populations while sparing non-senescent counterparts, which has been shown to delay the aging process in murine models [61]. The discovery of senolytics has increased the potential for treating multiple age associated diseases simultaneously. Senescent cell clearance via senolytics may be an effective strategy to promote closure of chronic wounds, similar to their therapeutic potential in other chronic diseases. There are several subclasses of therapeutic agents that have been repurposed or used as senolytics. This includes BCL-2 inhibitors, flavonoids, and metformin amongst others that have been thoroughly reviewed [62,63,64,65,66,67,68,69]. 

Members of the BCL-2 protein family can act to either inhibit apoptosis in cells, thus promoting cell survival, or serve as pro-apoptotic agents [62]. In a typical cell, these BCL-2 partners will be balanced [62]. When anti-apoptotic pathway members predominate, the cell is arrested at the G0 phase of the cell cycle [62]. A BCL-2 inhibitory senolytic drug, Navitoclax, promotes apoptosis in a wide range of senescent cells via sequestration of anti-apoptotic BCL-2 family members, though it does not eliminate all senescent cells [13,70]. Navitoclax has limited range of impact on senescent cells due to heterogeneity in senescent cell populations which can rely on different anti-apoptotic pathways for survival [13,71]. Additionally, Navitoclax is associated with toxic side effects (e.g., thrombocytopenia and neutropenia) [13]. Navitoclax has not been applied to the treatment of chronic wounds yet, although another senolytics agent, UBX0101, has been used [72,73]. Studies have shown that the injection of UBX0101, into aged mice cleared senescent cells and reduced symptoms of osteoarthritis [72,73]. A clinical trial adapted UBX0101 treatment for osteoarthritis into humans but, was paused in phase II due to inability to outperform the placebo group [2]. 

The use of flavonoids is a common strategy to clear senescent cells from murine models [10,26]. Flavonoids are natural phenolic structures found in plants that are associated with beneficial impacts on health [63]. Flavonoids are known to be antioxidative, anti-inflammatory, anti-mutagenic, and anti-carcinogenic with chemical properties that enhance key enzymatic functions and pathways [63]. Two common flavonoids used in senescence research are Quercetin (Q) and Fisetin (F) [2,13]. Although it exerts senolytic activity when used alone, the flavonoid Quercetin is typically coupled with Dasatinib, a SRC/tyrosine kinase inhibitory chemotherapeutic agent [13]. This combination therapy is used to exploit synergistic effects that target multiple senescent cell associated anti-apoptotic pathways (SCAPs) [13]. SCAPs are redundant pro-survival pathways present in senescent cells, which downregulate key apoptotic modulators (e.g., caspases) [2,13]. This has the added benefit of broadening the therapeutic index, meaning the treatment has a broader efficacy with fewer side effects [13].

Metformin is a commonly used therapeutic for the treatment of diabetes mellitus and has been used since the 1950′s [65]. This drug has since garnered interest in the field of aging research given its association with decreased all-cause morbidity and mortality [66]. Metformin has senolytic and senotherapeutic activity through inhibition of the SASP and SCAPs [13]. In a 2010 study, metformin was used on the invertebrate *Caenorhabditis elegans* and promoted a longer lifespan with an increased health span [67,68]. This was also demonstrated in murine models [68]. Metformin is currently being investigated in the Targeting Aging with Metformin (TAME) clinical trial which aims to expand its indication to more broadly target age associated disease [74]. The TAME trial aims to compare patients being treated with metformin with patients who have stopped metformin treatments [74,75]. The occurrence of different age-related pathologies is then compared between each group of participants [74,75].

As discussed previously, senescent cells and the associated SASP can play different roles in different wound types. For example, certain senescent cell populations play a beneficial role in acute wound healing, so eliminating them could be detrimental to the healing of acute wounds. Further research is needed to investigate therapies that can be used to alleviate senescent cell load without disrupting the overall healing process.

## 8. Discussion 

As discussed, senescent cutaneous cells involved in wound healing operate differently in an acute and chronic setting, which is partially attributable to differential SASP signaling in these contexts. A chronic wound SASP promotes defective epithelial barrier formation and excessive fibrosis [76]. However, it is important to note that much of the work implicating cellular senescence in wound healing comes from murine models, which bear translational caveats [76]. In particular, wounds in mice heal primarily via contraction of the panniculus carnosus, which is not present in humans. For future investigation into the heterogeneity of chronic and acute wounds, a model that resembles human skin will need to be adopted for validation [76]. Evidence points toward using swine as a model for wound healing as their skin has the closest resemblance to humans [76]. Investigating wound healing in a more closely related model will allow for more direct translational findings. 

In addition to these inherent caveats of current wound healing model systems, there are a number of key unanswered questions regarding the role of cellular senescence in wound healing. For example, it is unclear if senescent cell accumulation varies between wounds that heal via primary vs. secondary intention mechanisms or similarly how accumulation kinetics depend upon wound thickness. Furthermore, it is incompletely understood how senescent cell burden is regulated (i.e., are senescent cells cleared from wound sites primarily via intrinsic induction of apoptosis or via immune mediated clearance) and whether this changes with wound chronicity. While not limited to the field of wound healing, senescent cell profiling is largely informed by transcriptomic datasets and proteomic insight is limited. This is in part due to current technologic limitations with single cell proteomic characterization techniques, especially those that can capture signatures of low abundance cell types such as senescent cells. This is an area of great interest given that it is unknown how the transcriptome and proteome of senescent cells correlate, which is of particular concern for aging tissues [77]. Improved “omics” based insight would also help to address to what degree senescent cell phenotypes vary from their non-senescent counterparts. For example, as macrophages are heavily implicated in both physiologic and pathological cutaneous wound healing processes, it is important to define whether senescent macrophage subtypes fulfill different roles in wound closure than those without senescent features. 

Defining a consistent marker that is present in senescent cells (e.g., fibroblasts or macrophage populations) may reveal new therapeutic opportunities for chronic wounds that feature dysregulated SASP cues. Unfortunately, there are few effective therapeutic options for treatment resistant chronic wounds in the clinic. With an improved understanding of senescent cell heterogeneity and the roles of these cells in wound healing, senolytic drugs hold promise as therapies for many senescence related conditions including non-healing wounds and diabetic ulcers. As stated previously, there is difficulty in distinguishing subtypes of senescent cells. This is in part due to limitations in isolating pure senescent cells from tissues [16]. This constrains the ability to define the function of senescence in normal, aged, or injured tissues and is further complicated by the underlying heterogeneity of senescent cells. A better understanding of this heterogeneity and characterization of discrete senescent cell subpopulations could vastly improve the prognosis of associated diseases. Identifying biomarkers for these populations, including distinct cell markers, SASP members, and SCAP components could help to make for effective and specific therapies that are able to target deleterious senescent cell populations while sparing those that exert beneficial effects. Furthermore, examining how senescent cell populations contribute to tissue repair within pathologic contexts, such as chronic wounds, may promote the general knowledge of fields such as regenerative medicine. 

## 9. Conclusions

Overall, cellular senescence has been implicated with multiple facets of the wound healing process. Cell populations involved in wound healing including vascular, immune, and mesenchymal components adopt senescence features and possess a senescence related secretome. However, acute and chronic wounds vary in their senescent cell composition which is further confounded by the underlying heterogeneity of these populations within each wound type, which likely relates to their differential clinical presentations. Identifying specific roles that the major senescent cell populations play in chronic and acute contexts may allow for tailored senotherapies that preserve the beneficial effects of the SASP on wound resolution while limiting wound-prolonging SASP features. Experimental cutaneous wound models are attractive systems to study senescence given that both the homeostatic and pathologic roles of senescence biology can be appreciated in a timely manner compared to the study of senescence in chronologically aging systems. Findings from these models may evoke new regenerative therapies and an improved appreciation of the biology of cellular senescence.

## Figures and Tables

**Table 1:** Senescent cells present in acute cutaneous wounds listed alongside how they contribute to tissue repair.

Senescent Cell Type	Role in Healing Process	Reference
Fibroblasts	Secrete PDGF-AA * in SASP * which helps differentiation of non-senescent fibroblasts	[54]
Myofibroblasts	Limiting fibrosis through ECM *-degrading-SASP *	[55]
Endothelial	Promote wound closure through secretion of PDGF-AA * in SASP *	[18,54]
Macrophages	Inflammation response, debris resolution, tissue remodeling	[42]

* Platelet-derived growth factor alpha polypeptide a (PDGF-AA); Senescence-associated secretory phenotype (SASP); Extracellular Matrix (ECM).

## Data Availability

Not applicable.
